# THE VALUE OF PREOPERATIVE PROGNOSTIC NUTRITIONAL INDEX IN GASTRIC CANCER AFTER CURATIVE RESECTION

**DOI:** 10.1590/0102-6720202400012e1805

**Published:** 2024-06-17

**Authors:** Francisco TUSTUMI, Marina Alessandra PEREIRA, André Safatle LISAK, Marcus Fernando Kodama Pertille RAMOS, Ulysses RIBEIRO, André Roncon DIAS

**Affiliations:** 1 Universidade de São Paulo, Hospital das Clínicas, Instituto do Câncer do Estado de São Paulo, Department of Gastroenterology – São Paulo (SP), Brazil.

**Keywords:** Stomach Neoplasms, Gastrectomy, Nutrition Assessment, Survival, Neoplasias do Estômago, Gastrectomia, Avaliação Nutricional, Sobrevivência.

## Abstract

**BACKGROUND:**

Predicting short- and long-term outcomes of oncological therapies is crucial for developing effective treatment strategies. Malnutrition and the host immune status significantly affect outcomes in major surgeries.

**AIMS:**

To assess the value of preoperative prognostic nutritional index (PNI) in predicting outcomes in gastric cancer patients.

**METHODS:**

A retrospective cohort analysis was conducted on patients undergoing curative-intent surgery for gastric adenocarcinoma between 2009 and 2020. PNI was calculated as follows: PNI=(10 x albumin [g/dL])+(0.005 x lymphocytes [nº/mm^3^]). The optimal cutoff value was determined by the receiver operating characteristic curve (PNI cutoff=52), and patients were grouped into low and high PNI.

**RESULTS:**

Of the 529 patients included, 315 (59.5%) were classified as a low-PNI group (PNI<52) and 214 (40.5%) as a high-PNI group (PNI≥52). Older age (p=0.050), male sex (p=0.003), American Society of Anesthesiologists score (ASA) III/IV (p=0.001), lower hemoglobin level (p<0.001), lower body mass index (p=0.001), higher neutrophil-lymphocyte ratio (p<0.001), D1 lymphadenectomy, advanced pT stage, pN+ and more advanced pTNM stage were related to low-PNI patient. Furthermore, 30-day (1.4 vs. 4.8%; p=0.036) and 90-day (3.3 vs. 10.5%; p=0.002) mortality rates were higher in low-PNI compared to high-PNI group. Disease-free and overall survival were worse in low-PNI patients compared to high-PNI (p<0.001 for both). ASA III/IV score, low-PNI, pT3/T4, and pN+ were independent risk factors for worse survival.

**CONCLUSIONS:**

Preoperative PNI can predict short- and long-term outcomes of patients with gastric cancer after curative gastrectomy. Low PNI is an independent factor related to worse disease-free and overall survival.

## RESUMO

**RACIONAL:**

Estimar os desfechos de curto e longo prazo das terapias contra o câncer é crucial para o desenvolvimento de estratégias de tratamento eficazes. A desnutrição e o estado imunológico do hospedeiro afetam significativamente os desfechos em cirurgias de grande porte.

**OBJETIVOS:**

Avaliar o valor do índice nutricional prognóstico pré-operatório (INP) na predição de desfechos em pacientes com câncer gástrico.

**MÉTODOS:**

Foi realizada uma análise de coorte retrospectiva de pacientes submetidos à cirurgia com intenção curativa para adenocarcinoma gástrico entre 2009 e 2020. O INP foi calculado da seguinte forma: INP=(10 x albumina [g/dL])+(0.005 x linfócitos [nº/mm^3^]). O valor de corte ideal foi determinado pela curva característica de operação do receptor (ponto de corte do INP=52), e os pacientes foram agrupados em INP baixo ou alto.

**RESULTADOS:**

Dos 529 pacientes incluídos, 315 (59,5%) foram classificados como grupo de baixo INP (INP<52) e 214 (40,5%) como grupo de alto INP (INP>52). Idade mais avançada (p=0,050), sexo masculino (p=0,003), escore da Sociedade Americana de Anestesiologistas (ASA) III/IV (p=0,001), menor nível de hemoglobina (p<0,001), menor índice de massa corpórea (p=0,001), maior relação neutrófilos-linfócitos (p<0,001), linfadenectomia D1, estágio pT avançado, pN+ e estágio pTNM mais avançado foram relacionados ao paciente com baixo INP. Além disso, as taxas de mortalidade em 30 dias (1,4 vs. 4,8%; p=0,036) e em 90 dias (3,3 vs. 10,5%; p=0,002) foram maiores no grupo com baixo PNI em comparação ao grupo com alto INP. A sobrevida livre de doença e a sobrevida global foram piores em pacientes com baixo INP em comparação com pacientes com alto INP (p<0,001 para ambos). Escore ASA III/IV, baixo INP, pT3/T4 e pN+ foram fatores de risco independentes para pior sobrevida.

**CONCLUSÕES:**

O INP pré-operatório pode predizer desfechos de curto e longo prazo de pacientes com câncer gástrico após gastrectomia curativa. Baixo INP é um fator independente relacionado a piores sobrevida livre de doença e sobrevida global.

## INTRODUCTION

Gastric cancer (GC) is a significant health concern worldwide, characterized by its low survival rates due to its frequent diagnosis at advanced stages^17,31^. Surgery remains the primary curative option for most patients^16^. However, gastrectomy is associated with a significant risk of postoperative complications and mortality. Over 20% of patients experienced substantial postoperative morbidity, and the 30-day mortality rate was around 4%^24^.

The risk of surgical complications is especially relevant in cancer due to preoperative nutritional deterioration commonly found in GC patients. The obstructive nature of gastric neoplasms leads patients undergoing gastric resection to experience prolonged periods of reduced caloric and protein intake before surgery^3^. Furthermore, cancer stimulates the production of inflammatory interleukins, as indicated by increased serum inflammatory markers in cancer patients. This inflammatory condition contributes to a worse prognosis for the patient and an increased risk of postoperative complications^37,38,41,42^.

In this setting, having a straightforward preoperative strategy to stratify patients at risk for poor postoperative outcomes is crucial for improving patient selection for gastric resection. A clear preoperative strategy can help identify patients likely to benefit most from surgery and those requiring additional support or interventions before the procedure. By identifying high-risk patients early on, healthcare providers can implement pre-habilitation programs to optimize patients’ physical and nutritional status, potentially reducing postoperative complications and improving outcomes^34^.

Considering the close relationship between malnutrition and host’s immune status and postoperative outcomes, preoperative markers of nutrition and systemic inflammation are crucial for stratifying risk in GC patients. The prognostic nutritional index (PNI) is a straightforward marker of nutrition status and systemic inflammation. This index is based on serological routine tests, calculated as PNI=(10 × albumin [g/dL])+(0.005 × lymphocytes [nº/mm^3^]). Thus, this study aimed to evaluate the significance of preoperative PNI as a predictor of short- and long-term outcomes in GC patients.

## METHODS

### Study design

A retrospective cohort was conducted on patients submitted to gastrectomy in a single cancer institute. An experienced surgical oncology team performed all surgeries. The surgical technique was performed according to the Japanese Gastric Cancer Association guidelines^16^ and the Brazilian Gastric Cancer consensus^4^.

### Eligibility

Patients with GC who underwent potentially curative gastrectomy between 2009 and 2020 were included. Only patients with histologically proven gastric adenocarcinoma and D1 or D2 lymphadenectomy were selected. Exclusion criteria comprised metastatic disease, gastric remnant tumors, infection, emergency surgery, or incomplete medical records.

### Preoperative workup

Data were collected prospectively from a database. All patients were submitted to clinical and anesthesia preoperative evaluation. Patients underwent blood tests, endoscopy, and computed tomography (chest and abdominal) up to one month before surgery. Preoperative PNI was defined as PNI=(10 × albumin [g/dL])+(0.005 × lymphocytes [nº/mm^3^]). Tumors were staged according to the TNM staging system from the eighth edition of the American Joint Committee on Cancer manual^2^.

### Data extraction

The following data were extracted: blood test results, computed tomography reports, age, sex, body mass index (BMI), the extent of lymphadenectomy, type of resection, tumor size, histological type, lymphatic invasion, venous invasion, perineural invasion, American Society of Anesthesiologists (ASA) preoperative risk score, and Charlson comorbidity index (CCI), without age and neoplasia in the score.

Follow-up was performed every three months in the first year and every six months after this period, with a clinical evaluation. Studies to detect relapse were performed based on the presence of symptoms.

### Outcomes

Postoperative complications were classified using the Clavien-Dindo scale. Clavien-Dindo ≥ 3a were considered major complications^12^. We evaluated 30- and 90-day mortality rates after surgical resection. Overall survival (OS) and disease-free survival (DFS) were estimated based on the interval from surgery to death, recurrence, or the last contact.

### Statistical analysis

The data were described as mean and standard deviation (SD) for quantitative variables and absolute and relative frequencies for qualitative variables.

The receiver operating characteristic (ROC) curve with area under the curve (AUC) was plotted to evaluate the ability of PNI to predict 90-day mortality. The optimal cutoff value was determined by the maximum Youden index, and patients were grouped into “low-PNI” and “high-PNI” groups. Pearson’s chi-square (χ²) test, Student t-test, or the Mann-Whitney U test were used for comparisons.

The Kaplan-Meier curve was used to analyze survival, and the Log-rank test was used to compare the groups. Multivariate cyclooxygenase (Cox) proportional hazard analysis was performed to determine independent risk factors for survival. Only variables significant in univariate analysis (p<0.050) were selected for the multivariate analysis.

A significance level of 5% was considered, and the analyses were performed using the Statistical Package for Social Sciences (SPSS), v20.0 software (IBM Corp., 2016).

This study was approved by the local Ethics Committee, which waived the consent form (CAAE: 43247321.0.0000.0068).

## RESULTS

**Figure 1:**
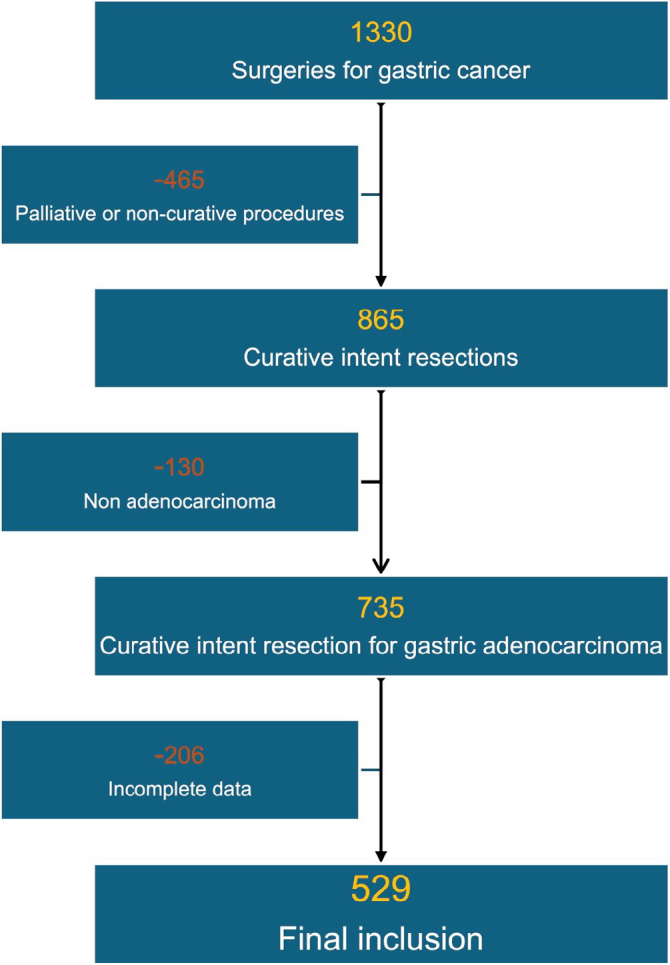
Patients’ selection flow diagram.

Of the 1,330 surgeries performed, 529 patients met the inclusion criteria and were evaluated in this study. Figure 1 shows the flow diagram with the patients’ selection. The mean age was 62.8 years (range 22–94), and 59.5% were male. Subtotal gastrectomy and D2 lymphadenectomy were performed in 64.1% and 82.4% of patients, respectively. Stage III was the most common (43.1%), and 55.5% of patients had lymph node metastasis (pN+).

**Figure 2:**
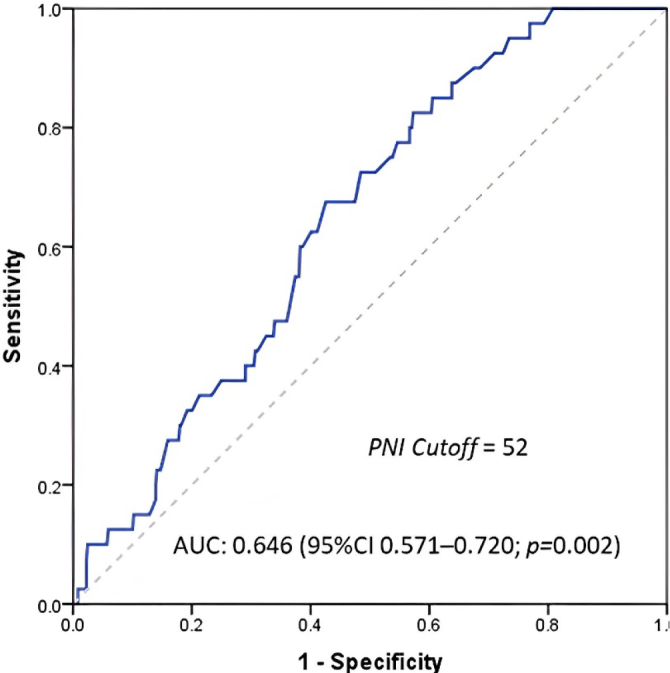
A receiver operating characteristic curve was performed to predict 90-day mortality according to the prognostic nutritional index value.

The average PNI value was 50.1 (SD±14.7), and the median was 50.5 (IQR 45.5–54.0). The performance metric for PNI was assessed by constructing the ROC curve (Figure 2). The AUC for the PNI score was 0.646 (95%CI 0.571–0.720; p=0.002), and the optimal cutoff value was 52.

Thus, based on the cutoff value determined by the ROC curve, 315 (59.5%) patients were classified as a low-PNI group (PNI<52); and 214 (40.5%) as a high-PNI group (PNI>52). The clinical and surgical characteristics of both groups are presented in Table 1.

**Table 1:** Clinical and surgical characteristics according to the prognostic nutritional index.

Variables	High PNI	Low PNI	p-value
	n=214 (%)	n=315 (%)	
Sex			
Female	103 (48.1)	111 (35.2)	0.003
Male	111 (51.9)	204 (64.8)	
Age (years)			
Mean (SD)	60.9 (12.7)	64.1 (12.7)	0.005
Body mass index (kg/m²)			
Mean (SD)	25.3 (4.5)	23.9 (4.8)	0.001
Charlson comorbidity index			
CCI 0	140 (65.4)	200 (63.5)	0.650
CCI ≥1	74 (34.6)	115 (36.5)	
ASA classification			
I/II	175 (81.8)	217 (68.9)	0.001
III/IV	39 (18.2)	90 (31.1)	
Hemoglobin (g/dL)			
Mean (SD)	13.2 (1.7)	11.5 (2.2)	<0.001
NLR			
Mean (SD)	1.87 (1.1)	3.46 (3.3)	<0.001
Lymphadenectomy			
D1	23 (10.7)	70 (22.2)	0.001
D2	191 (89.3)	245 (77.8)	
Type of resection			
Subtotal	145 (67.8)	194 (61.6)	0.147
Total	69 (32.2)	121 (38.4)	

PNI: prognostic nutritional index; SD: standard deviation; CCI: Charlson comorbidity index; ASA: American Society of Anesthesiologists score; NLR: neutrophil-to-lymphocyte ratio.

Older age (p=0.050), male sex (p=0.003), ASA III/IV (p=0.001), lower hemoglobin level (p<0.001), lower BMI (p=0.001), higher neutrophil-lymphocyte ratio (p<0.001) and D1 lymphadenectomy were more common in the low-PNI group.

Regarding pathological characteristics (Table 2), the low-PNI group had larger tumors (p<0.001) and a higher rate of venous (p<0.001) and perineural invasion (p=0.037) compared to the high-PNI group. Furthermore, patients with low PNI were associated with more advanced pT stage (p<0.001), presence of lymph node metastasis (p=0.033), and more advanced pTNM (p=0.004).

**Table 2:** Pathological characteristics and postoperative outcomes according to the prognostic nutritional index.

Variables	High PNI	Low PNI	p-value
	n=214 (%)	n=315 (%)	
Tumor size (cm)			
Mean (SD)	3.7 (2.1)	5.5 (3.3)	<0.001
Histological type			
Intestinal	113 (52.8)	189 (60.0	0.101
Diffuse	101 (47.2)	126 (40.0)	
Histological differentiation			
Well/moderate	95 (44.4)	162 (51.4)	0.112
Poor	119 (55.6)	153 (48.6)	
Lymphatic invasion	91 (42.5)	158 (50.2)	0.084
Venous invasion	51 (23.8)	126 (40)	<0.001
Perineural invasion	87 (40.7)	157 (49.8)	0.037
pT			
T1/T2	118 (55.1)	108 (34.3)	<0.001
T3/T4	96 (44.9)	207 (65.7)	
No retrieved lymph nodes			
Mean (SD)	42.4 (19.5)	40.1 (16.9)	0.151
pN			
pN0	107 (50.0)	128 (40.6)	0.033
pN+	107 (50.0)	187 (59.4)	
pTNM			
I/II	137 (64.0)	162 (51.4)	0.004
II/IV	77 (36.0)	153 (48.6)	
Length of hospital stay			
Mean (SD)	11 (9.0)	13.5 (10.9)	0.007
Postoperative complications			
No/Minor	189 (88.3)	263 (83.5)	0.122
Major	25 (11.7)	51 (16.5)	
30-day mortality	3 (1.4)	15 (4.8)	0.036
90-day mortality	7 (3.3)	33 (10.5)	0.002

cm: centimeter; SD: standard deviation; PNI: prognostic nutritional index; pN: lymphonodes staging; pTNM: tumor staging.

The length of hospital stay was lower for the high-PNI group than low-PNI (11.0±9.0 vs. 13.5±10.9; p=0.007). There was no significant difference in postoperative complications between the two groups (p=0.122). The 30-day (1.4 vs. 4.8%; p=0.036) and 90-day (3.3 vs. 10.5%; p=0.002) postoperative mortality rate was lower in the high-PNI group. There was no significant difference in the administration of adjuvant chemotherapy between the high-PNI and low-PNI groups (49.5 vs. 51.4%, respectively; p=0.669).

### Survival analysis

**Figure 3:**
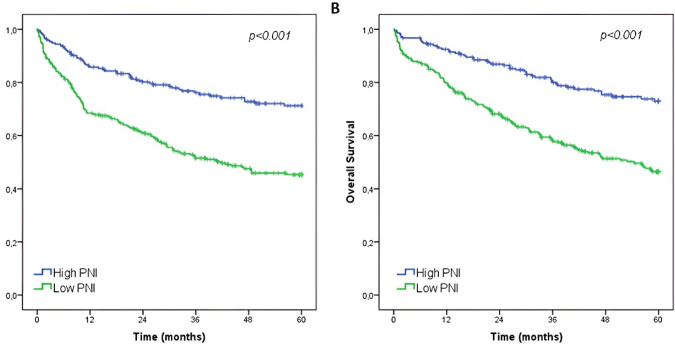
Disease-free survival and overall survival according to prognostic nutritional index groups.

The median follow-up was 36.8 months, 119 patients had recurrence, and 194 died during the follow-up. The estimated 5-year OS for the entire cohort was 57.3%. Low-PNI patients had a worse DFS and OS compared to the high-PNI group (estimated 5-year DFS: 71.2 vs 45.3%, p<0.001; estimated 5-year OS: 72.9 vs 46.4%, p<0.001) (Figure 3).

In multivariate analysis, ASA III/IV score, low PNI, total gastrectomy, advanced tumor stage (pT3/T4), and presence of positive lymph nodes (pN+) were independent risk factors for worse DFS and OS in GC patients. Table 3 shows the univariate and multivariate analyses.

**Table 3:** Univariate and multivariate analysis for disease-free survival and overall survival.

Disease-free survival	Univariate			Multivariate		
Variables	HR	95%CI	p*	HR	95%CI	p*
Male (vs. female)	1.50	1.13–1.99	**0.006**	1.19	0.89–1.59	0.254
Age ≥65 (vs. <65 years)	1.26	0.97–1.65	0.089	–	–	–
Charlson ≥2 (vs. 0–1)	1.27	0.97–1.68	0.086	–	–	–
ASA III/IV (vs. ASA I/II)	1.90	1.42–2.53	**<0.001**	1.66	1.24–2.22	**0.001**
Low PNI (vs. high PNI)	2.37	1.74–3.22	**<0.001**	1.95	1.43–2.67	**<0.001**
Total gastrectomy (vs. subtotal)	1.65	1.25–2.16	**<0.001**	1.42	1.08–1.86	**0.012**
pT3/T4 (vs. pT1/T2)	2.97	2.16–4.08	**<0.001**	1.83	1.27–2.62	**0.001**
pN+ (vs. pN0)	3.01	2.20–4.10	**<0.001**	2.07	1.46–2.95	**<0.001**
non-CT (vs. CT)	1.06	0.81–1.39	0.661	–	–	–
**Overall survival**	**Univariate**			**Multivariate**		
**Variables**	**HR**	**95%CI**	**p** *	**HR**	**95%CI**	**p** *
Male (vs. female)	1.54	1.14–2.07	**0.005**	1.23	0.91–1.67	0.172
Age ≥65 (vs. <65 years)	1.39	1.05–1.85	**0.021**	1.45	1.09–1.92	**0.011**
Charlson ≥2 (vs. 0–1)	1.23	0.93–1.65	0.152	–	–	–
ASA III/IV (vs. ASA I/II)	1.93	1.43–2.61	**<0.001**	1.69	1.25–2.28	**0.001**
Low PNI (vs. high PNI)	2.47	1.79–3.42	**<0.001**	1.95	1.40–2.72	**<0.001**
Total gastrectomy (vs. subtotal)	1.61	1.21–2.14	**0.001**	1.44	1.08–1.92	**0.014**
pT3/T4 (vs. pT1/T2)	2.96	2.17–4.15	**<0.001**	1.90	1.29–2.79	**0.001**
pN+ (vs. pN0)	1.85	2.06–3.93	**<0.001**	1.93	1.33–2.70	**<0.001**
non-CT (vs. CT)	1.11	0.84–1.47	0.469	–	–	–

* p-values in bold are statistically significant.

HR: hazard ratio; CI: confidence interval; CT: adjuvant chemotherapy; ASA: American Society of Anesthesiologists; PNI: prognostic nutritional index; pT1/T2/T3/T4: tumor staging; pN0: lymphonodes staging; pN: lymphonodes staging.

## DISCUSSION

Inflammation and malnutrition are critical factors in the prognosis of GC. In this setting, PNI is a valuable marker, providing insights into the impact of the host’s immune system status and malnutrition on surgical outcomes. Our findings suggest that preoperative lymphocyte reduction, alongside a protein deficiency, is associated with a higher risk for postoperative mortality and decreased survival.

This study showed that low PNI values are associated with worse histopathological prognostic variables, such as venous and perineural invasion, pN, and pT. These results indicate that PNI might have a significant role in cancer progression and dissemination, and consequently, PNI can depict the tumor’s aggressiveness. These biological features of bad prognosis eventually promote poor long-term survival rates. Other studies also showed that PNI might have a significant impact on survival rates in endometrial, ovarian, and esophageal cancer^18,27,46^.

In addition, our study showed that PNI was associated with age, ASA classification, extent of lymphadenectomy, and postoperative mortality, which implies that PNI also reveals patients’ vulnerabilities and overall clinical status. Consequently, PNI presents a global picture of the patient and the tumor and can be a valuable tool for GC patients’ risk stratification. In our center, gastrectomy with D1 dissection is generally performed in elderly or frail patients with low-performance status and comorbidities, which is what was observed in the low-PNI group^30,36^.

Patient stratification is crucial in planning preoperative strategies for individuals undergoing surgery. By identifying patients at risk of poor outcomes, healthcare providers can implement tailored interventions^32^. Prehabilitation programs, which include nutritional support, exercise, and psychological interventions, can help improve at-risk patients’ physical and mental resilience before surgery^15^. Nutritional support, such as oral supplements or enteral nutrition, can help correct nutritional deficiencies and improve overall health status^43^. Stratifying patients allows for personalized care plans that address specific needs, ultimately reducing the risk of complications, postoperative mortality, and longer survival rates^9^.

The tumor-associated inflammatory response reflects the host’s immune status and antitumor immune response^14^. Inflammation plays a significant role in cancer development and progression, affecting tumor initiation, promotion, and metastasis^10,25^. Cancer cells release growth factors and inflammatory mediators that stimulate the production of peripheral leucocytes, producing factors that disrupt the tumor stroma, facilitating invasion and metastasis^1,22^. Tumors attract inflammatory cells, including macrophages and lymphocytes, which produce cytokines and chemokines^40^. Chronic inflammation creates a tumor-promoting microenvironment by releasing inflammatory mediators, growth factors, and cytokines, stimulating cell proliferation, angiogenesis, and resistance to cell death. Inflammation also suppresses the immune response against tumors, allowing cancer cells to evade immune surveillance^8^. Lymphocytes play a crucial role in eliminating neoplastic cells, and lymphopenia weakens the antitumor immune response, increasing the probability of tumor dissemination^8,25^.

As a result, persistent inflammation is associated with a poorer prognosis in various types of cancer, including gastric cancer. Inflammatory markers, such as the neutrophil-to-lymphocyte ratio (NLR), platelet-to-lymphocyte ratio (PLR), and C-reactive protein (CRP) levels, have been identified as prognostic indicators in numerous cancer types^6,21,26,33,44,45^.

High levels of these markers are often associated with more aggressive tumor behavior, higher rates of recurrence, and poorer survival outcomes, as seen in previous studies with GC patients undergoing jejunostomy, stage IV GC, and patients with multivisceral resections^11,28,29^. Previous studies have highlighted the relevance of systemic markers of systemic inflammation markers on gastric and esophagogastric cancer prognosis. Szor et al.^38^ evaluated the role of NLR, a systemic inflammation biomarker, on gastric cancer prognosis. The authors found that NLR was associated with lower survival rates, higher depth of tumor invasion, and positive nodal involvement. Tustumi et al.^41^ studied esophageal cancer patients undergoing neoadjuvant chemoradiotherapy followed by esophagectomy. They found that lymphocytes decreased during neoadjuvant therapy and predicted severe postoperative complications. In addition, a high NLR was associated with a higher risk for recurrence and low survival rates.

Albumin is commonly used as a nutritional assessment marker before surgery due to its widespread availability and relatively low cost. It serves as an indicator of nutritional status, reflecting long-term dietary intake and protein synthesis^35^. Low albumin levels have been linked to increased morbidity and mortality in surgical patients, making it a valuable prognostic indicator. In addition, albumin also plays a role in depicting patients’ systemic inflammation^13^. Albumin levels can decrease during inflammatory states due to increased capillary permeability and redistribution. Despite its convenience, albumin has limitations. Its levels can be affected by factors beyond nutrition or inflammation, such as liver disease and hydration status, reducing specificity^7^. Consequently, in addition to albumin levels, a comprehensive nutritional evaluation should include a precise clinical assessment and measures of sarcopenia^19^. Clinical evaluation involves assessing factors such as weight loss, dietary intake, and physical function, which can provide valuable insights into a patient’s nutritional status. Measures of sarcopenia, such as muscle mass and strength assessments, can help identify patients at risk of poor surgical outcomes and guide preoperative interventions^5^. Combining these assessments with traditional nutritional markers like albumin levels can provide a more holistic understanding of a patient’s nutritional status and help tailor preoperative strategies to improve outcomes.

Malnutrition is a significant risk factor for adverse outcomes in patients undergoing cancer resection surgeries, including an increased risk of mortality^20^. Malnutrition weakens the immune system, impairs wound healing, and reduces the body’s ability to withstand the stress of surgery, leading to an increased risk of postoperative complications. Malnourished patients are more likely to experience surgical site infections, delayed wound healing, and prolonged hospital stays, all of which contribute to an increased risk of mortality. Additionally, malnutrition can exacerbate the catabolic state induced by surgery, leading to further muscle wasting and functional decline^23^.

The current study has limitations. The dynamic nature of blood cellular components and albumin levels, which may vary daily in the same patient, and the influence of factors other than systemic inflammation and malnutrition should be considered. Besides, numerous approaches exist for determining optimal cutpoints for PNI. The choice of cutpoint in continuous variables can influence p-values, leading to the acceptance or rejection of null hypotheses. We determined the cutpoint based on the Youden index in ROC curves for the outcome “90-day mortality”. This choice has some drawbacks since time-to-event outcomes, such as OS and DFS, might not be precisely discretized according to the Youden index^39^.

Larger, controlled prospective studies are warranted to validate the predictive value of these inflammatory markers in GC prognosis. Indeed, future research is necessary beyond validating inflammatory markers in GC prognosis. Developing nomograms and prognostic calculators could significantly enhance the ability to determine the best treatment strategy for patients with GC. These tools could integrate various clinical, pathological, and inflammatory markers and nutritional status indicators to assess the patient’s condition comprehensively. Additionally, future trials should incorporate prehabilitation and preoperative nutritional support according to risk stratification estimation, optimizing patient outcomes by addressing malnutrition and enhancing physiological reserves before surgery.

## CONCLUSIONS

Preoperative PNI can predict short- and long-term outcomes of patients with GC after curative gastrectomy, and low PNI is an independent factor related to worse DFS and OS. Also, low-PNI patients have poor clinical conditions, advanced pathological stage, and high postoperative mortality compared to those with high-PNI. These findings underscore the importance of considering inflammatory markers and nutritional status in managing GC patients, with the potential to improve risk stratification and treatment outcomes.
